# The Hamburg Youth Prevention Project (HYPP) for adolescents with sexual interest in children

**DOI:** 10.1038/s41443-023-00755-9

**Published:** 2023-08-26

**Authors:** Fabiola Casademont, Viktoria Märker, Carola Bindt, Peer Briken

**Affiliations:** 1 Institute for Sex Research, Sexual Medicine, and Forensic Psychiatry, University Medical Center Hamburg-Eppendorf, Hamburg, Germany; 2 Department of Child and Adolescent Psychiatry, University Medical Center Hamburg-Eppendorf, Hamburg, Germany

**Keywords:** Therapeutics, Health care

## Abstract

Paedophilic sexual interest is often linked to an emergence during adolescence, but concrete empirical knowledge on its development and early viable treatments remains scarce. The aim of this article is to provide an overview about the current state of research on juveniles with sexual interest in children as well as to introduce the Hamburg Youth Prevention Project (HYPP). The HYPP aims to better comprehend the development of sexual interests in adolescents and seeks to find a basis for improved treatment strategies during this critical developmental period. In this perspective, we outline the framework, goals, and treatment approach for this project. It addresses adolescents who are sexually attracted to younger children or engage in sexual acts with children and who have not yet been in contact with the justice system. The project offers a diagnostic process, anonymous counselling, and treatment. It is grounded in a biopsychosocial perspective on psychosexual development and an integrative family-centred approach. The project is based on the core assumption that in adolescent participants, there is still great flexibility for development, including their sexual interests.

## Addressing divergent sexual interests in adolescence

Puberty and adolescence are highly vulnerable developmental phases characterised by neurobiological and physical changes, as well as changes in motivation, cognition, behaviour, and social relationships [1]. Along with these changes, physical and psychological development of sexual identity, orientation and interests occur, which can pose a considerable challenge for juveniles [2]. Especially the discovery of divergent sexual interests can be highly challenging to adolescents. This discovery might involve recognising a diversified sexual orientation or paraphilic, e.g., paedophilic interests. Some of these adolescents could also be at risk of acting on their interests, i.e., child sexual abuse or consumption of child sexual abuse images.

The purpose of this perspective is to call attention to the fact that there is a group of adolescents, who are sexually attracted to or engage in sexual acts with significantly younger children, and who have not yet been in contact with the justice system. The difficulties in diagnosis and the underrepresentation of this population in current research are summarised, and a respective treatment programme, the Hamburg Youth Prevention Program (HYPP), is presented.

### Diagnosing paedophilic disorders in adolescence

Regarding research on adults with paedophilic disorders, evidence suggests that in retrospect, paedophilic interests become recognisable during adolescence [3–5]. However, forward-looking, a diagnosis of a paedophilic disorder during adolescence entails difficulties: According to the International Classification of Mental Diseases [6], a paedophilic disorder is characterised by a “sustained, focused, and intense pattern of sexual arousal—as manifested by persistent sexual thoughts, fantasies, urges, or behaviours—involving pre-pubertal children”. Individuals either act on these impulses or experience them as markedly distressing. The DSM-5 [7], altogether similar in its criteria, further specifies that the individual must be at least 16 years old and at least 5 years older than the children they are attracted to.

These main criteria, however, cannot be fully applied to adolescents. The age criterion of being at least 16 years old does not apply to all individuals with a sexual interest in children [4]. Furthermore, the sustainability and persistence of sexual interest in children have yet to be awaited: Little is known about how paedophilic interests arise in adolescence, what their characteristics are, and how they typically develop. Whereas some researchers argue that paedophilic interest represents a life-long condition [8], there is also evidence to suggest that such interests might change [9]. Due to the lack of consistent data, it has not yet been clarified for whom this interest might be more or less flexible.

For our programme, one assumption is that in adolescence, there is still great flexibility in development, also concerning sexual interests. In light of the aforementioned unknown variables, we will use the more comprehensive term “sexual interest in (younger) children” rather than “paedophilia or paedophilic disorder” in this paper. This takes into account a variety of possible manifestations of the symptomatology in adolescents.

## The importance of early prevention

While the mere sexual attraction to (much younger) children is not illegal, some affected adolescents might feel distressed because of their interest or might even be at risk of acting on their interests and therefore committing an offence. Research shows that a substantial part of sexual offences is committed by youth: In Germany, children and juveniles under the age of 18 account for 12.8% of the suspects for sexual coercion, assault, and rape [10]. Since it is likely that a certain percentage of adolescents committing sex offences (ASOs) are never reported and therefore never prosecuted by the law, it is to be expected that the official statistics underestimate the number of sexual offences committed by adolescents. This seems especially important considering the results of a representative survey showing that victims are reluctant to report sexual offences [11]. Considering these figures, a treatment approach is necessary to prevent sexual abuse at an early stage.

In this paper, the term “undetected” will be used to refer to adolescents who have not yet been in contact with the justice system. This category includes those who have participated in criminal activities that have remained unreported, hence never reaching the attention of law enforcement or judicial systems, and consequently have not been subject to prosecution.

### Adolescents committing sexual offences

A meta-analysis of studies comparing ASOs to adolescents who have committed non-sexual offences revealed several factors linked to their criminal behaviour. ASOs were more likely to have atypical sexual interests, which accounted for the largest group difference, more likely to have been victims of child sexual abuse themselves as well as of other forms of abuse or neglect, more likely to have experienced social isolation, and low self-esteem [12]. Also, ASOs had fewer delinquent peers, fewer substance abuse problems, and more limited criminal histories. However, most of the studies only included convicted and institutionalised ASOs [12], which makes it difficult to translate the findings to undetected adolescents.

There also seem to be different possible triggers as to why adolescents commit sexual offences, e.g. a lack of knowledge or inexperience with sexuality [13]. Furthermore, some adolescents seem to commit sexual offences when they are only vaguely recognising their interests and still discovering their own sexuality [14]. These examples show that factors associated with child sexual abuse committed by adolescents could be preventable through early counselling and treatment [13]. Hence, preventive approaches addressing the onset of sexually harmful behaviour and targeting juveniles only beginning to exhibit sexual problem behaviour are almost non-existing [15] but of utmost importance.

### Emotional response to acknowledging a sexual interest in children

Also, irrespective of sexual offending, even acknowledging a sexual interest in children is accompanied by displeasing emotions, such as uncertainty, guilt, and self-hatred [4]. Research showed that this often led to social isolation and emotional as well as physical distancing from peers [4]. Individuals also state that the negative affect and the fear of stigmatisation are intensified through negative views by media and the general public [4, 14]. These results show that preventive treatment to help adolescents with a sexual interest in children or those at risk to refrain from offending cannot be postponed until adulthood.

## Treatment of adolescents who have sexually offended

The efficacy of treatment of ASOs is supported by research [16–19]. In particular, cognitive-behavioural and multisystemic approaches show great promise [16, 18, 19]. However, most treatment studies base on juveniles who already sexually offended and are known to the police and are accordingly charged and convicted for the offence [16]. While treatment guidelines are available for this subgroup [16], studies examining the treatment of undetected juveniles—therefore taking possible selection effects into account [20]—are scarce. Trying to fill this gap, in contrast to after-the-fact interventions for offenders or preventive methods targeting potential victims [15], we present a prevention programme that specifically targets adolescents with a sexual interest in younger children, who claim not to want to act on their interest and who have not yet been in contact with the justice system (i.e. being undetected)—regardless of whether they have ever acted according to this preference. As stated above, numerous variables concerning sexual interest in children during adolescence remain unknown. This leads us to also include (young) adolescents in our project who may not (yet) express an enduring sexual interest in children but have already engaged in sexual acts with children, as their sexual preferences may not be fully understood at this stage.

## The Hamburg Youth Prevention Project (HYPP)

Given the lack of well-established early intervention programmes for the assigned group of undetected adolescents, the HYPP is still exploratory in nature. The project’s central aims are:

1. developing and establishing a low-threshold, anonymous primary care programme for adolescents with sexual interests in children,
2. preventing child sexual abuse and minimising the risk for adolescents to commit sexual offences,
3. an enhanced knowledge of the development of sexual interest in children and
4. providing and improving health care to maintain and stabilise the psychosocial and sexual health of at-risk adolescents.

Thus, the overall long-term goal is minimising negative health consequences on an individual and societal level and increasing the knowledge about this specific group of adolescents. These health consequences concern potential victims’ mental as well as physical health [21] but also the adolescents’ psychological and emotional well-being [4]. On a societal level, preventive treatment of individuals potentially committing sexual offences could reduce the cost of prosecution and possible incarceration after an offence took place.

The HYPP offers a diagnostic process, anonymous counselling, and psychotherapy as well as medical treatment to those who are at risk for (further) child sexual abuse because they are sexually attracted to children or committed child sexual abuse. Financing is covered by the health insurance system (GKV) in a model project, which makes it possible for juveniles to attend the project but remain anonymous towards their health insurance company. The anonymity lowers the threshold for participation, as individuals have to worry less about stigmatisation early in life. German law also allows medical confidentiality towards the justice system regarding past offences (§203 German Penal Code).

The diagnostic and therapeutic procedure and selection of risk-assessment instruments are based on the guidelines for treating ASOs with paraphilic disorders of the *World Federation of Societies of Biological Psychiatry* [16].

### Inclusion in the project

Anyone interested may contact the programme via telephone or email. Information about the project can be obtained directly from the University Hospital website (https://www.uke.de/kliniken-institute/institute/institut-fuer-sexualforschung-sexualmedizin-und-forensische-psychiatrie/behandlungsangebot/praeventionsambulanz/index.html) and was also distributed to educational institutions, the police, victim and offender contact points, and child and adolescent psychiatric in- and outpatient services. It is important to note that, up to now, juveniles rarely approach the HYPP at their own request, but rather because a significant adult found out about the sexual interests or sexual activities of the patient that inflicted harm on others or were cause for concern. This might be due to a slow process of awareness of the adolescent’s sexual interests deviating from others, or lacking the language to describe deviant attraction [4], because individuals do not consider them distressing enough to seek treatment, or have strong feelings of shame [16]. As a result, treatment motivation is often ambivalent, which is addressed early and repeatedly throughout therapy. Additionally, the patients are frequently motivated by the fear of adverse outcomes if they choose not to participate, such as being suspended from school or having their parents confiscate their mobile phone.

Between April 2018 and December 2022*, N* = 95 inquiries reached the project, of which one female and 37 male adolescents aged 12–18 years participated or are still participating. In rare cases, younger people from the age of 8 also sought help. We did not include *n* = 57 individuals mainly due to not meeting the inclusion criteria: being sexually interested in children/having committed child sexual abuse, not being in contact with the justice system (i.e., being undetected), no severe psychiatric comorbidities (acute psychosis, substance abuse), no severe cognitive impairment, age being 12–18 years (whereas younger patients were admitted in exceptional cases). Reasons for inclusion in the project were, e.g., sexual interest in younger children, hands-on sexual abuse, consumption of child sexual abuse images, encouraging children to engage in mutual sexual acts, as well as requesting/sending pictures of their genitalia to younger children.

To determine if sexual interest in children or sexual acts with children are considered harmful, clinicians must rely on their professional judgement. In this project, considerations are made for whether the behaviour occurs without the other party’s consent (or if the other party is even capable of giving consent) and whether a noticeable age gap exists. The evaluation also takes into account the sexual, emotional, and physical maturity of the child and adolescent [16], as well as any potential power imbalances that may arise from these factors. Also, in alignment to legal sanctions for adults (§173 German Penal Code), we take into consideration if the sexual acts with children have an incestuous component. Furthermore, the distress caused by sexual interest in children is taken into account.

### Diagnostic phase

The initial diagnostic sessions focus on investigating the issues and events that led to the presentation in the project, both with the parent or legal guardian and with the adolescent alone in single-session clinical interviews. From 16 years of age, participation is also possible without parents or guardians. The interviews focus on the medical and psychological history as well as on psychosexual development (see Table 1 for an overview). Also, standardised assessment of psychopathology, personality traits, behavioural problems, and competencies take place by administering questionnaires to the participants and their parents/legal guardians (see Table 2 for an overview). The therapists also regularly complete standardised risk assessment instruments, which are specifically designed to address the dynamic nature of risk factors in adolescents: the Juvenile Sex Offender Assessment Protocol-II [22] and the Estimate of Risk of Adolescent Sexual Offense Recidivism, Version 2.0 [23].

**Table 1:** Clinical interview during diagnostic phase.

Focus of assessment
Medical and psychological history
Multiaxial child and adolescent psychiatric diagnostic
Somatic diseases
Prior treatments
Suicidality
Patient’s psychosocial environment
Family and personal history of psychiatric comorbidities, e.g., ADHD, addictive disorders
Psychosexual development
Family sexual socialisation (sexuality education, dealing with sexuality in the family)
Pubarche and development of gender and sexual identity
(Non-)deviant sexual fantasies and their exclusiveness
Age of onset of paraphilic fantasies or behaviours
Masturbatory practices and fantasies including use of pornography
Own sexual experiences (e.g., not limited to but including playing sexual discovery games, witnessing sexual activities of others, first kisses, or other experiences)
Sexual abuse

**Table 2:** Overview of standardised diagnostic instruments used in the HYPP, adapted from [35].

Instrument	Construct measured
For the patient	
PSSI [37]	Inventory of personality styles and disorders: Assessment of personality variables
YSR/11-18R [38]	Youth self-report: Behavioural problems, emotional problems, somatic complaints, and social skills
DISYPS-III [39]	Diagnostic self-assessment for psychiatric disorders in children and adolescents according to the ICD-10 and DSM-5
CTS [40]	Childhood abuse and neglect
Diagnostics of social competence [41]	Questionnaire of social competences for children and adolescents
MSWS-ESS [42]	Assessment of self-esteem
UCLA-LS [43]	Assessment of social isolation
E-Scale [44]	Assessment of the capacity for empathy
PPJ-SDS-S [45]	Current sexual behaviours: Self-assessment
For the parent, legal guardian, caregiver	
CBCL/6-18R [38]	Child Behaviour Checklist: Third party assessment matching the YSR/11-18R
DISYPS-III [39]	Diagnostic third-party assessment for psychiatric disorders matching the DISYPS-III patient version
For the therapist	
J-SOAP-II [22]	Juvenile Sex Offender Assessment Protocol-II: Assessment of risk of recidivism
ERASOR 2.0 [23]	Estimate of Risk of Adolescent Sexual Offense Recidivism
PPJ-SDS-F [45]	Current sexual behaviours: Third party assessment

For all questionnaires, German versions are used.

### Treatment

The HYPP is based on a biopsychosocial understanding of sexuality [24] and a specialised form of treatment aligned with the *World Federation of Societies of Biological Psychiatry* guidelines [16]. The treatment follows the risk-need-responsivity principles [25], where the treatment intensity is derived from the degree of risk for sexual problem behaviour. Regarding the need principle, the focus lies on dynamic risk factors (derived from the standardised risk assessments used), including, e.g., deviant sexual fantasies, attachment difficulties, or deficits in affect regulation and social skills. Responsivity factors concern patient characteristics, e.g., whether the adolescent’s language skills or the motivation for treatment are sufficient. An individual disorder hypothesis is developed together with the patient. This hypothesis often has to be revised when working with adolescents since strong fluctuations in the patient’s further (psychosexual) development occur. This need for revision also applies to therapy goals agreed upon at the beginning, which must be adjusted repeatedly.

The psychotherapeutic treatment in the HYPP usually lasts 1–2 years and takes place once a week in an outpatient setting. The therapy is not strictly manualised but generally consists of biographical work, work on dynamic risk factors, and behavioural change [26]. An individualised therapeutic programme is preferable to a rigid manual to treat the heterogeneous group properly [27]. Medication may be offered as needed, e.g. SSRIs to control intense, sexual impulses or urges [28]. Following a multi-systemic approach, in addition to individual sessions and treatment, parents/guardians and institutions are often also included in the treatment.

A common treatment goal is to assist the patient in making sense of the functionality and meaning of the (abusive) sexual attitudes or acts, e.g., striving for social attention, overcoming power imbalances, deviant sexual interests, seeking attachment and contact, feeling safe, or experimenting with physical arousal. Rendering meaning to the behaviour may also serve as a means to deal with shame and strengthen the patient-therapist working alliance. A psychodynamic understanding of symptoms can also be helpfully applied (however, mostly, without using psychodynamic treatment techniques).

With regards to the techniques, the HYPP uses sexuality education (e.g., explanation of physiological reaction and arousal patterns in adolescents, differences between what can be considered as appropriate and non-appropriate sexual behaviour) and mainly cognitive behavioural treatment methods, including reducing cognitive distortions, dealing with atypical sexual arousal, and identifying individual warning signs that potentially lead to (further) offending. Furthermore, active and empathic questioning is necessary to invite young people to talk about their thoughts and feelings. At times, young people also find it easier to express their emotions and fantasies through playing together.

### Obstacles in sex therapy with adolescents

When conducting sex therapy involving adolescents, practitioners must navigate different obstacles. For example, adolescents often have difficulties putting their problems and feelings into words [29], as the capacity to mentalise their feelings and reflect upon them is still maturing [30, 31]. Therefore, the therapeutic dialogue may be perceived as stress. Also, asking about the patient’s psychosexual development and sexual history requires professional expertise and reflectiveness on the therapist’s part. Pronounced vagueness, justifications, or denial often characterise conversations about sexuality. To reduce the risk of misunderstandings and facilitate communication, it is helpful to rephrase and repeat any questions in case of uncertainties, using developmentally appropriate language.

## Possible indicators of treatment success

As the primary measure of successful treatment, one commonly looks at the risk of recidivism [27]. Additionally, studies have shown different indicators of therapeutic change as a measure for successful treatment of individuals committing sex offences, such as enhancing locus of control, or accepting responsibility for offences [32, 33]. However, whether therapeutic changes in these variables translate into reduced recidivism remains unclear [33, 34].

Measuring successful treatment in the HYPP, which includes cases where no previous offences took place and only the sexual interest itself was the reason for presentation, presents challenges. As recidivism risk is difficult to measure and so far, there has been no systematic research regarding other proximal indicators of treatment success among these adolescents, we conceptualised “successful” treatment in the HYPP in broader terms: In addition to the self-reported absence of sexual offences and refraining from using child sexual abuse images, we also consider it a success if patients stated that they comprehend possible psychosocial stressors and risk factors, avoid corresponding behaviours, and learn alternative behaviours. Furthermore, we regard it as a success if patients express an understanding of their sexual fantasies and underlying related factors (e.g., compensating for a power imbalance, experiencing emotional closeness to others). It remains to be investigated whether these factors also have a measurable impact on a reduced onset of sexual abuse/likelihood of recidivism.

## Improving treatment offers

Exploring the perspective of adults suffering from paedophilia can provide valuable insights for developing effective treatment strategies: Research showed that individuals with paedophilic interests, when asked as adults, aimed for positive role models, positive messaging, and general support. They also explicitly described the wish for non-judgmental therapeutic services, expanding knowledge about sexual interest in children, strengthening relationships with parents, and addressing deficits in sexuality education [4]. These needs should be considered when implementing new treatment modalities.

As outlined above, the HYPP presents a novel treatment approach targeting adolescents who have sexual interests in children or have engaged in sexual acts with children and are undetected. To our knowledge, so far, there are only two other institutions in Germany offering a similar approach [35, 36]. Otherwise, there seem to exist only educational, school-based approaches [13], and online services for individuals with sexual interest in children, the latter offering support for all age groups (e.g. https://www.helpwantedprevention.org/). However, no systematic research has been conducted to identify risk and protective factors for this distinct group of undetected adolescents. As evident from the previous discussions, conducting scientific research in this area comes with numerous challenges. Despite this, it is essential to systematically examine institutions and their approaches to establish valid therapeutic techniques.

## Implications for future research

Further investigations should also address questions such as the reasons behind child sexual abuse occurrences and the risk and protective factors for child sexual abuse among undetected adolescents. Furthermore, differentiating between individuals with and without a sexual interest in younger children will aid in comprehending the intermediary role that sexual interest in children plays in child sexual abuse during adolescence. This research will foster a more comprehensive understanding of the complexities involved and yield better treatment options that align with patients’ needs and concerns.

## Conclusion

In summary, the HYPP highlights the crucial need to address and treat undetected adolescents at risk for committing child sexual abuse. Both adolescents and their parents express appreciation for the opportunity to participate in the project, particularly valuing the anonymity and the non-stigmatising approach. This had made discussing sexual interests in children much easier for the adolescents. It is essential to remain attentive to the insights of affected adolescents and incorporate this feedback into empirically-based, effective treatment strategies.
